# UK quantitative WB-DWI technical workgroup: consensus meeting recommendations on optimisation, quality control, processing and analysis of quantitative whole-body diffusion-weighted imaging for cancer

**DOI:** 10.1259/bjr.20170577

**Published:** 2017-12-07

**Authors:** Anna Barnes, Roberto Alonzi, Matthew Blackledge, Geoff Charles-Edwards, David J Collins, Gary Cook, Glynn Coutts, Vicky Goh, Martin Graves, Charles Kelly, Dow-mu Koh, Hazel McCallum, Marc E Miquel, James O’Connor, Anwar Padhani, Rachel Pearson, Andrew Priest, Andrea Rockall, James Stirling, Stuart Taylor, Nina Tunariu, Jan van der Meulen, Darren Walls, Jessica Winfield, Shonit Punwani

**Affiliations:** 1 Centre for Medical Imaging, University College London,University College London, London, UK; 2 Institute Nuclear Medicine, University College London Hospitals NHS Foundation Trust,University College London Hospitals NHS Foundation Trust, London, UK; 3 Clinical Oncology, Mount Vernon Cancer Centre, Northwood, UK; 4 Cancer Research UK Cancer Imaging Centre, Division of Radiotherapy and Imaging, Institute of Cancer Research,Institute of Cancer Research, Sutton, UK; 5 Medical Physics, Division of Imaging Sciences and Biomedical Engineering, Guy’s and St Thomas’—NHS foundation Trust, King’s College London, Guy’s and St Thomas’—NHS foundation Trust, King’s College London, London, UK; 6 School of Imaging Sciences and Biomedical Engineering, King’s College, London, UK; 7 MRI Unit, The Royal Marsden Hospital Foundation Trust, Surrey, UK; 8 Department of Radiology, Guy’s and St Thomas’ – NHS foundation Trust, London, UK; 9 MR Physics, The Christie NHS Foundation Trust, The Christie NHS Foundation Trust, Manchester, UK; 10 Department of Radiology, Cambridge University Hospitals NHS Foundation Trust,Cambridge University Hospitals NHS Foundation Trust, Cambridge, UK; 11 Department of Radiology, Northern Centre for CancerCare, Newcastle upon Tyne Hospitals, NHS Foundations Trust,Northern Centre for CancerCare, Newcastle upon Tyne Hospitals, NHS Foundations Trust, Newcastle upon Tyne, UK; 12 Department of Radiology, The Royal Marsden Hospital Foundation Trust,The Royal Marsden Hospital Foundation Trust, Surrey, UK; 13 Medical Physics, Barts Health NHS Trust, London, UK; 14 Division of Cancer Sciences, University of Manchester, Manchester, UK; 15 Department of Radiology, The Christie NHS Foundation Trust,The Christie NHS Foundation Trust, Manchester, UK; 16 Paul Strickland Cancer Centre, Mount Vernon Cancer Centre, Northwood, UK; 17 Northern Institute for Cancer Research, Newcastle University, Newcastle upon Tyne, UK; 18 PET Centre, King’s College, London, UK; 19 Department of Radiology, University College London Hospitals NHS Foundation Trust,University College London Hospitals NHS Foundation Trust, London, UK; 20 Department of Health Services Research and Policy, London School of Hygiene and Tropical Medicine,London School of Hygiene and Tropical Medicine, London, UK; 21 Institute Nuclear Medicine, University College London, London, UK

## Abstract

**Objective:**

Application of whole body diffusion-weighted MRI (WB-DWI) for oncology are rapidly increasing within both research and routine clinical domains. However, WB-DWI as a quantitative imaging biomarker (QIB) has significantly slower adoption. To date, challenges relating to accuracy and reproducibility, essential criteria for a good QIB, have limited widespread clinical translation. In recognition, a UK workgroup was established in 2016 to provide technical consensus guidelines (to maximise accuracy and reproducibility of WB-MRI QIBs) and accelerate the clinical translation of quantitative WB-DWI applications for oncology.

**Methods:**

A panel of experts convened from cancer centres around the UK with subspecialty expertise in quantitative imaging and/or the use of WB-MRI with DWI. A formal consensus method was used to obtain consensus agreement regarding best practice. Questions were asked about the appropriateness or otherwise on scanner hardware and software, sequence optimisation, acquisition protocols, reporting, and ongoing quality control programs to monitor precision and accuracy and agreement on quality control.

**Results:**

The consensus panel was able to reach consensus on 73% (255/351) items and based on consensus areas made recommendations to maximise accuracy and reproducibly of quantitative WB-DWI studies performed at 1.5T. The panel were unable to reach consensus on the majority of items related to quantitative WB-DWI performed at 3T.

**Conclusion:**

This UK Quantitative WB-DWI Technical Workgroup consensus provides guidance on maximising accuracy and reproducibly of quantitative WB-DWI for oncology. The consensus guidance can be used by researchers and clinicians to harmonise WB-DWI protocols which will accelerate clinical translation of WB-DWI-derived QIBs.

## INTRODUCTION

Whole body MRI, including diffusion-weighted imaging (DWI), offers significant advantages over other cancer imaging modalities; combining a high soft tissue contrast and adaptable spatial resolution with “functional” imaging without exposure to ionising radiation. Fuelled by recent technological advances, the use of whole body MRI in oncology is rapidly increasing, both for clinical research and for routine clinical imaging of specific indications (*e.g.* multiple myeloma).^1^ Whole body DWI (WB-DWI) enables assessment of RECIST non-measurable disease foci such as bone metastases^2^ and, as well as depicting anatomy, DWI provides microstructural information^3^ that can be correlated with tissue metabolism.^4^

Quantitative evaluation of DWI provides a measure of the average apparent diffusion coefficient (ADC) of water within a voxel. Differences in ADC between voxels reflect differences in the cellular composition (microstructure) of individual voxels. Despite many recent publications demonstrating the potential of ADC to act as a quantitative imaging biomarker (QIB) for oncology,^5–11^ WB-DWI-derived ADC assessment has not become widely used in clinical practice, and has struggled with adoption as the primary endpoint biomarker in multicentre trials. Key factors limit generalizability of WB-DWI, these include: (i) the complexity of optimising WB-DWI protocols to produce artefact free images; (ii) the lack of standardization of WB-DWI acquisition parameters; and (iii) heterogeneity in derivation and interpretation of ADC values.^12^

To address these factors and promote research and clinical applications of WB-DWI, there is a need to agree hardware and software requirements, and provide sequence optimization, acquisition, reporting and quality control (QC) guidance for maximising accuracy and reproducibility.^11–16^ To address this challenge, a group of experts (UK Quantitative WB-DWI Technical Workgroup) was convened from cancer centres around the UK with subspecialty expertise in quantitative imaging and/or the use of WB-DWI within clinical and/or research practice.

This document is the output from this group and is intended to act as guidance for clinicians, radiographers and MR physicists/clinical scientists who are considering development/implementation of quantitative WB-DWI (qWB-DWI) for clinical trial or routine clinical use. The main recommendations from this review are listed in Tables 1–3.

**Table 1.** ****+, agree but no consensus; *, agree with consensus; **, strongly agree with consensus

*Harmonisation*	
One acquisition protocol should be created to cover all quantitative (response) assessment of metastatic disease (“one size fits all”)	*
Define Matrix size	+
Define Slice thickness	*
Define fat suppression technique	*
Define minimum number of *b*-values	*
Define min/max *b*-values	*
Whole body (head to mid-thigh)	*
It should be possible to do quantitative (response) assessment on data from different scanners of the same manufacturer/model	**
It should be possible to do quantitative (response) assessment on data from different scanners of different manufacturers	*
An appropriately trained person should always perform a set up optimisation with a suitable test object.	**
A regular QC test should be carried out with a suitable test object.	**
It should be possible to define a post-processing pipeline that allows any ADC map from any scanner to be compared.	**

ADC, apparent diffusion coefficient; QC, quality control; **

**Table 2.** Bolded items 1.5T and 3T, Consensus result in columns for routine clinical/multi-centre trial. +, agree but no consensus; *, agree with consensus; **, strongly agree with consensus. Italics indicate round table consensus

	Clinical	Trial
*Acquisition Protocol*		
**Do not use intrinsic body coil as a receiver coil**	**	**
**Axial from cranial vertex to mid-thigh**	**	**
**Total acquisition time < 30 mins**	**	**
***Free breathing***		
In-plane acquisition pixel size ≥ 3 × 3 mm (matrix 128 × 128 FoV ~ 380 × 420)	*	*
Minimum slice thickness = 5 mm	*	*
**40–50 slices per bed station**	*	*
**Single shot EPI (Contiguous slices, interleaved)**	**	**
***Parallel imaging (Reduction factor = 2) ***		
Fat suppression method = STIR (160 ms <TI < 180 ms)	*	**
***Minimum number of b-values = 2 ***		
***b*-values = 50–100 s mm^–^**^**2**^**, 800–1000 s mm^–^**^**2**^	*	*
**Diffusion weighted images: trace weighted **	+	+
Number of averages = 3 (more at higher *b* value if possible, dependent on grad encode scheme)	+	*
*TE < 95 ms *		
*TR ≥ 5×T1 of tissue of interest*		
*Optimisation Protocol*		
**Always perform initial optimisation on suitable test object**:	*	**
**minimally diffusing**, (*or doped water*)	*	**
**known ADC values**,	*	**
**controlled temperature **(*e.g*. iced water phantom)	*	**
**larger than total number of slices **(per bed station)	*	**
**Minimise the**:		
**Static field distortion**	*	**
**eddy-current induced distortion **	*	**
**ghosting**	**	**
**Optimise fat suppression on human volunteers**	*	**
**Assess repeatability and reproducibility for multi-centre trials on human volunteers**	+	**
*Routine Quality Assurance*		
**Routine QC measurements should be performed and** **recorded**	**	**
**Not necessary to test daily or weekly**	+	+
**every 3 months**	*	*
**after software/hardware upgrades**	**	**
**after repairs/maintenance**	*	*
**Routine Quality control measurements should be performed with a suitable test object: **		
**iced water**	+	*
**sugar/(*****doped*****)water at room temperature**	+	*
**Routine Quality control tests**		
**ADC linearity in *Z*-direction across all slices in bed station**	+	*
Static field distortion	+	*
**Eddy-current induced distortion**	+	*
**Ghosting**	+	*

ADC, apparent diffusion coefficient; FOV, field of view; TE, echo time; TR, repetition time; QA, quality assurance; QC, quality control; STIR, short tau inversion-recovery.

**Table 3.** Bolded items staging and response assessment. +, agree but no consensus; *, agree with consensus; **, strongly agree with consensus

*Processing and Analysis*	
**Apply SNR threshold before calculating ADC**	+
**Calculate ADC map on each bed station separately before stitching**	*
**Voxel-wise mono exponential/no *b* = 0 model**	*
**Do not use bi-exponential or stretched exponential model**	**
Use VOIs to extract ADC values from multiple lesions	+
**Use low *b*-value image to delineate VOI**	*
**Do not extract histogram characteristics**	*
**Do not use absolute thresholding to define VOI/ROI**	*
*Visualisation and Reporting*	
**Report mean ADC**	*
**Report median ADC**	*
**Report SD of ADC**	+
**Report volume of VOI**	+
Report alongside other modalities (US, CT, PET)	+
**Do not use RECIST 1.1 inspired reporting**	*
**Report % change in mean/median ADC values**	*
**View ADC maps of each bed station separately**	+
**View ADC maps as whole body images**	*
**View ADC maps in all 3 directions**	*
**View ADC maps alongside *b*-value images**	*
**View ADC maps alongside T2w and T1w images**	*
**ADC maps should not be fused with T2w, T1w images**	*
**High *b*-value signal intensity images should be viewed using a rotating Maximum Intensity Projections (MIPs)**	+
**Not necessary to produce a >*b* =1000 s mm^–^**^**2**^**image**	*
**View previous ADC maps for longitudinal studies**	*
**Use rigid body image registration for longitudinal studies**	+

ADC, apparent diffusion coefficient; FOV, field of view; QA, quality assurance; QC, quality control; STIR, short tau inversion-recovery; TE, echo time; TR, repetition time; STIR, short tau inversion-recovery.

## METHODS AND MATERIALS

### The consensus method

A consensus approach was developed based on the RAND/UCLA Appropriateness Method. This approach aimed to obtain consensus agreement regarding best practice for the implementation of qWB-DWI (http://www.rand.org/pubs/monograph_reports/MR1269.html). This method includes a combination of remote and face-to-face consensus rounds and combines the best available scientific evidence with the collective judgment of experts to yield statements regarding the appropriateness of relevant aspects of the topic under investigation. It is particularly suited to areas with a relative paucity of high quality level 1 evidence (*e.g.* randomized controlled trials) or, if the evidence is available, it does not contain sufficient detail to guide practice applicable to the range of patients seen in everyday clinical practice.

When using this method, appropriateness levels are used to communicate the perceived balance between risks/costs and benefits of each item under discussion. Our approach followed the RAND/UCLA Appropriateness Method as it is set out in the user’s manual throughout the process, as much as possible.^17^

### Panel selection

Leading clinicians, radiographers and scientists from the UK, with known subspecialty expertise in quantitative imaging and/or the use of imaging to inform treatment, were approached (MR physics, MR radiology, nuclear medicine, radiotherapy physics, oncology). An independent chair was selected, with experience using formal consensus methods to develop clinical guidelines. A total of 25 panel members were confirmed.

### Construct of the questionnaire

An extensive questionnaire containing 369 items was constructed between August and November 2015. The first draft was produced by four panel members with a background in MR physics.

The questionnaire was split into six main areas for consideration—initially defining the scope and requirement for (i) harmonisation; followed by addressing specific items needing consensus to achieve harmonisation goals, specifically: (ii) hardware specification, (iii) optimization, (iv) routine quality assurance (QA), (v) acquisition parameters and (vi) analysis and visualisation requirements. Within each section questions differentiated requirements for multicentre trials versus routine clinical practice, and between 1.5 and 3T magnetic field strengths.

- *Harmonisation*: This section was structured to identify critical requirements for a qWB-DWI protocol, and thereby provide a common standard; extending from desired applications to analysis and personnel needed to implement the technique.
- *Hardware Specifications*: This section asked questions to ascertain the minimum specifications that an MR scanner must have in order to acquire qWB-DWI and how to deal with variability in MRI hardware across sites.
- *Site optimisation*: These questions were split into two groups; one for optimising a clinical service and the second for a trial site.
- *Routine QA*: These questions were split into two groups; one for routine QA and control for a clinical service and the second for a trial site.
- *Acquisition parameters*: This section lists some basic acquisition parameter ranges based on current practice in centres using qWB-DWI protocols. Topics included fat suppression technique, maximum and minimum *b*-values, slice thickness, in-plane voxel sizes, imaging bandwidth and number of signal averages.
- *Analysis, visualisation and reporting*: This section considers analysis of the images including the processing of the data, such as intensity thresholding or motion correction prior to the ADC calculation, as well as how the data is visualised in the most useful way for a radiologist to report.

### First-round questionnaire completion before the meeting

All panel members were sent the questionnaire as an online survey (29 February 2016 to completed by 24 March 2016), with relevant literature on the RAND/UCLA appropriateness method and technical articles on quantitative WB-DWI via the project website (https://sites.google.com/site/wbadcconsensus/home). They were instructed to score each item on a Likert scale^18^ between 1 (strongly agree) and 5 (strongly disagree). A midpoint score of 3 indicated not necessary (or it does not matter) and a further category 6 (do not know) was used for the member to indicate that they did not have sufficient expertise to answer the question.

The questionnaire responses were collected and summarised by the consensus co-ordinator. A complete list of questions and modal answers are listed in Appendix A. Cell is colour coded grey if no consensus was reached, the text indicates the value of the mode answer of the panel.

### Face to face meeting format

The meeting was convened for one day in London, 12 April 2016. 23 panelists attended (2 were unable to attend the face-to-face discussions). The co-ordinator convened the meeting and documented key points of discussion. The whole meeting was audio recorded in order to check points in the discussion when preparing the manuscript.

At the beginning of the consensus meeting, selected expert panel members presented on the following topics: DWI in oncology, QA in DWI and practical aspects of performing whole-body DWI. Speakers were asked to summarise the evidence in the given area and to highlight areas of controversy.

Thereafter, for each individual question included in the questionnaire, a summary of the panel scores was presented and the topic discussed by the panel. After the discussion, the panelists rescored that item and were free to maintain or change their original response from the prior on line completion stage.

Four questions were reworded during the panel discussion to improve clarity. Six questions were added and scored during the meeting. 12 questions were removed during the meeting. In particular, it was decided to remove questions 6 and 7 regarding the use of whole body imaging as a tool for the assessment of other diffuse disease such as inflammation, *i.e.* there was consensus on using this technique only for wide-spread oncological disease. Questions 57 and 63 were also removed, since it was felt the use of open bore magnets to measure quantitative WB-DWI was a moot point, *i.e.* there was consensus not to use this type of scanner. Questions 360–367 were removed owing to the panel’s general agreement that there was currently a lack of data regarding the usefulness of using percentage change in ADC values as a response criteria or at what point it should be measured post-treatment to be able to answer these questions, *i.e.* there was consensus that this should not be used as a criteria to report. Questions 94–97 regarding the tests to be performed during acquisition optimisation were added during the discussion. One more question was added to the routine QA/QC section to allow the option of performing QC test quarterly, and four items that included the title “clinical scientist” were changed to “appropriately trained personnel”. In all cases, these changes were made with full agreement of the members of the panel and scored during the meeting.

### Interpretation of the results

The results of the second round of scoring were interpreted according to the RAM user’s manual,^17 ^*i.e.* only those items scored (on the scale 1–5 and not 6) by at least eight panel members were included in the results (every single item met this criteria). Consensus was defined as described in the user manual and listed below in Table 4.

**Table 4.** Key to calculate how consensus is defined; such that if there are more than the number of answers in the second column that are not within the mode response then there is NO CONSENSUS. This translates roughly to 70–80% of agreement between responders

**Number of delegates**	**Number of delegates whose answer is outside the mode**
8–10	No more than 2
11–13	No more than 3
14–16	No more than 4
17–19	No more than 5
20–22	No more than 6
23–25	No more than 7

The modal answer is calculated for each question and then the number of answers outside of the 2-point range that includes the modal answer (agree score, 5–4; not necessary score, 3; disagree score, 2–1) are counted. If there are more than a certain number (Table 4 above) outside the 2-point range answer, then the question is deemed to have reached “no consensus” as stated in the RAND/UCLA manual p. 58^17^ for a scale of 1–9 where the corresponding 3 point ranges are agree, (1–3) not necessary (3–6) disagree (7–9).

## RESULTS

Supplementary material (Supplementary material available online.) includes the complete list of questions and modal scores before and after the panel discussion. Cells colour coded white indicate that consensus was reached and grey indicates that it was not. The text indicates the value of the mode answer within the group.

Consensus was achieved on 197/369 prior to and 255/351 following the consensus meeting. Tables 1–3 lists the items whose modal answer was agree (*) or strongly agree (**) with consensus or agree with no consensus (+). Areas for which consensus were not reached are listed in Table 5 where the modal answer not necessary =(o) or disagree = (x).

### Harmonisation

Table 1a highlights the consensus amongst the expert panel on the general *need for harmonisation* of MR protocols, optimisation, QC and post-processing of qWB-DWI studies.

Although aspirational, the ability to compare ADC values (without reference to the hardware or software by which it was acquired) was unanimously agreed as a goal of harmonisation. The panel defined the scope of the consensus as pertaining to the use of qWB-DWI for oncological imaging rather than the assessment of other diffuse diseases, such as arthritis. Answers to questions in each subsequent section pertain to the aspiration and scope as defined by the panel. On the particular question “a clinical scientist should perform the site set-up optimisation” the panel came to a mutual agreement that “clinical scientist” (a protected professional title) should, therefore, be replaced by “an appropriately trained person”. During further discussion, complete agreement was also reached on the fact that consistent and comprehensive training in specialist quantitative MRI techniques will result in high quality and consistent image quality. A national training program could provide recommendations based on this consensus paper and practical hands-on experience of: site set-up and optimisation; routine QA; acquisition/scanning protocols; patient set-up; quantitative metrics; and statistics.

### Hardware specification and acquisition protocol/optimisation protocol/routine QA

**Figure 1.**
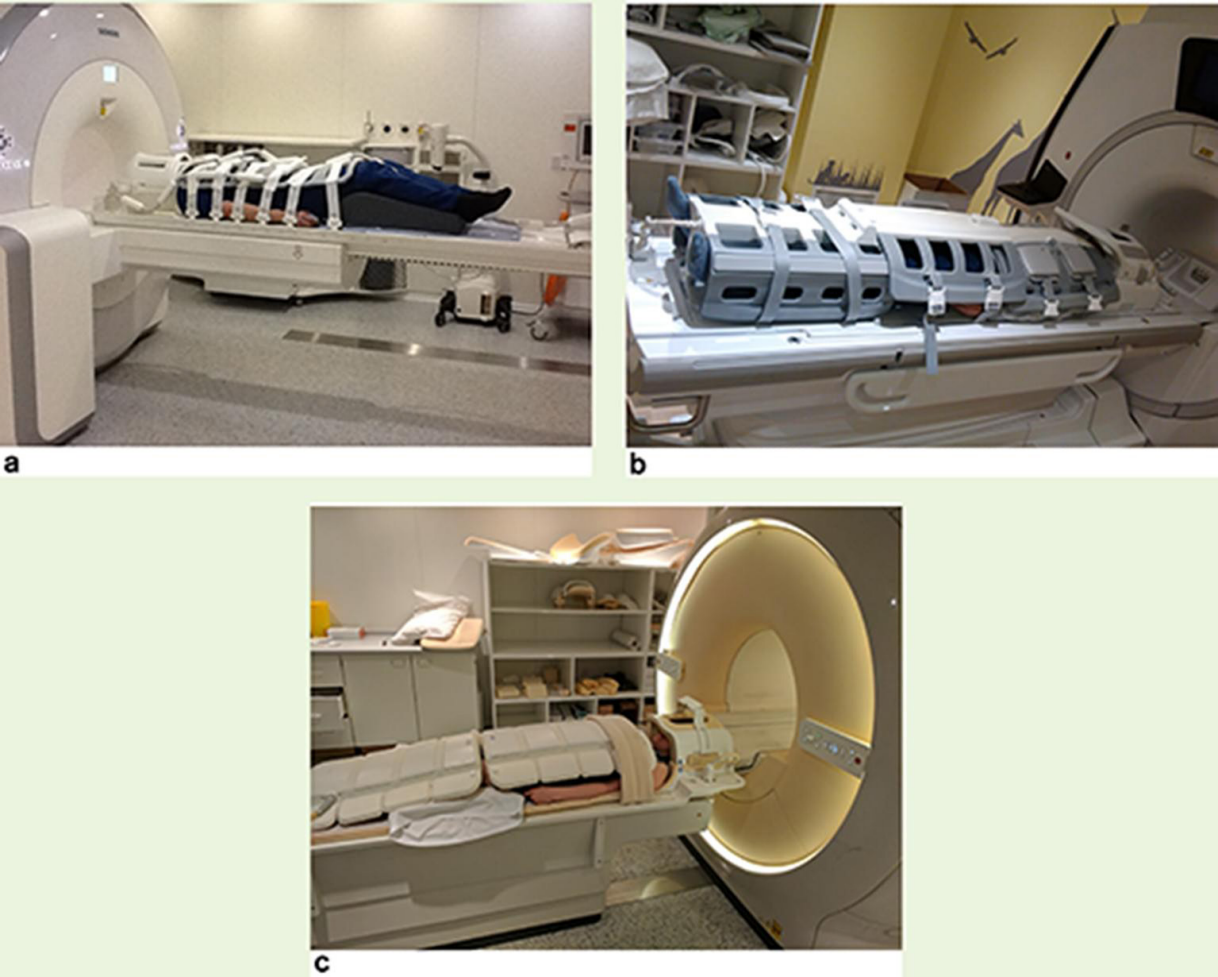
Typical patient set up for a whole body (eyes to thighs) MRI scan (a) Siemens Healthineers (Erlangen, Germany) (b) GE Healthcare (Waukesha, Wisconsin, USA) (c) Philips Healthcare (Koninklijke, The Netherlands).

Table 2 provides statements relating to consensus on specific acquisition parameters, optimization procedures and QA when setting up quantitative WB-DWI. Items that are bolded in Table 2 reached consensus at both 3T and 1.5T field strength for routine clinical and multicentre trial applications; non-bold items achieved consensus for 1.5T only for routine clinical scanning and multicentre trials. Figure 1 shows a typical whole body MRI scan patient set-up for the threee main scanner manufacturers.

#### Hardware specification and acquisition protocol 

The majority of the panel (those that were already familiar with acquiring qWB-DWI) felt strongly that robust qWB-DWI at 1.5T was achievable but it was acknowledged that (Table 5) there are challenges for translating these protocols to 3T platforms and that more evidence needed to be gathered on quantitative applications at 3T. In panel, consensus reflected previous recommendations,^12,19–22^ that WB-DWI is performed axially at multiple anatomical stations from head to midthigh (~4–5 sections) each acquired using the same MRI parameters and taking approximately 30 min in total. In order to acquire maximal SNR of DWI within a 30 min time-frame, this will typically limit scans to the acquisition of two *b*-values only: 50–100 and 800–1000 s mm^–^^2^. However, there are current scanner acquisition platforms such that *b* = 0 s mm^–^^2^ is collected by default in order to calculate ADC maps at the scanner console. There are, then, two options recommended by the panel; collect three *b*-values (at the expense of increased time for acquisition) where the second *b*-value should be between 50 and 200 s mm^–^^2^; collect two *b*-values (not *b* = 0 s mm^–^^2^) and perform the ADC calculation offline. Fat suppression should be used to remove unwanted signals from fat and multiple averages of each image to be acquired. Anecdotal evidence from panel members frequently using WB-DWI allowed an additional recommendation that the patient breathes freely during the diffusion-weighted acquisition. Basic acquisition parameters provided in Table 2 were agreed as a starting point for optimization at sites that have not previously performed WB-DWI. It was also agreed, that if not possible to implement even these basic parameters, then sites should not attempt quantitative WB-DWI, particularly for the purposes of multicentre trials.

**Table 5.** Summary of areas of no consensus. +modal answer agree; o, modal answer notnecessary; x, modal answer disagree;

Standardisation– No consensus	
Acquisition	
A minimum reconstructed pixel size at 1.5T and 3T	+
A minimum of 3 averages per *bb*-value at 1.5T and 3T for routine clinical	+
The gradient encoding scheme to be 3 orthogonal directions.	o
The fat suppression technique to be STIR at 3T only	o
The acquired slice thickness to be no less than 5 mm at 3T only	+
A maximum axial FoV at 3T	o
A minimum acquisition pixel size at 3T only	+
Optimisation	
No consensus for routine clinical imaging at 1.5T and 3T to	
Perform repeatability and reproducibility scans on normal volunteers	o
Perform optimisation of sequence on a sugared water test object at room temp	+
RoutineQA and QC	
No consensusfor routine clinical imaging at 1.5T and 3T to	
Perform monthly tests	o
The type of tests that should be done or how to do them	
ADC linearity in the *Z*-axis	+
Measure B0 distortion	o
Measure the eddy current distortion	o
ADC linearity in 3 different directions	+
What test object should be used	
Iced water phantom	+
With known biologically relevant ADC values	+
Minimallydiffusing medium (*e.g*. oil)	o
No consensus for research trial imaging at 1.5T and 3T to	
Performdaily or weekly tests	o
The type of tests that should be done	
ADC linearity in three directions	o
AnalysisVisualisation & Reporting	
No consensus was reached for basic ADC quantitation on whether	
A base signal intensity noise level should be set prior to ADCcalculation	+
To perform a bed station signal intensity normalisation over thewhole body prior to ADC calculation	x
No consensus was reached for disease staging and response assessment on how	
To define a ROI/VOI	
Geometric	o
Set threshold based on Max value	o
Use another MR weighted image to free draw VOI/ROI (staging only)	o
Whether whole body disease burden would be a useful measure	o
Reporting the standard deviation for each VOI/ROI	+
Specifying a percentage value of increase or decrease of tumour volume onsome ADC threshold to indicate positive or negative response respectively.	+
ADC values should be reported alongside	
Biopsy samples	o
Blood serum markers	o
Other modality images (response assessment only)	+
Using arotating greyscale MIP of whole body ADC map similar to whole PET scan	o
The need for image registration for longitudinal reporting	+

Although the panel supported the use of 3T for WB-DWI, it noted that there is currently insufficient evidence to recommend standardised basic acquisition protocols. Instead the panel recommends that a suitably trained person should carefully optimise all acquisition parameters listed in Table 2 for any particular 3T system.

#### Protocol optimisation:

Consensus recommendations for DWI as an oncology biomarker recommend that protocols should be “optimised to maximise SNR, minimise artefacts from ghosting and distortion and optimise fat suppression”.^11^ This panel also reached the same consensus. For multicentre trials, the panel recommended, as essential, protocol development using the parameters listed in Table 2 as a starting point, with the appropriate phantoms listed to interrogate the effects of eddy current-induced distortion and fat suppression. The reader is directed to an excellent review of these optimisation steps and their practical implementation described by Winfield et al^23^ and the latest IPEM report “Quality Control and Artefacts in Magnetic Resonance Imaging (Update of IPEM Report 80) – Report 112”.^19^

#### Routine QA: 

There was a clear distinction in number of QA items reaching consensus between routine clinical scanning and multicentre trials (3* vs *8 out of 10). While performing additional specific QA tests to support a multicentre trial was considered mandatory, there was no consensus on the need for additional specific QA in routine clinical practice. In general, the panel felt most MRI departments in the UK will have a regular routine QA strategies together with preventative maintenance contract with manufacturers/third party providers, which with optional additional coil checks on a daily/weekly basis (time and staff permitting) should be sufficient for routine clinical applications. The panel’s recommendation was to carry out routine QC tests listed in Table 2 (measurements for eddy current distortion, ghosting and ADC linearity in the *z*-direction) for both routine clinical and multicentre trials every 3 months and/or after major software or hardware upgrades or repairs. The panel cited the work by the American College of Radiology and the Association of American Physicists in Medicine (AAPM) on the practice and interpretation of regular QC tests and would seem a good place to start.^20,21^

### Processing and analysis/visualisation and reporting

Table 3 relates to statements where positive consensus was reached relating to processing, analysing, visualisation and reporting of quantitative WB-DWI. The items that are bolded and italicized in Table 3 are applicable to both staging and response assessment oncological applications, otherwise they are only applicable to response assessment.

#### Processing and analysis: 

This section achieved the least number of items of consensus both before and after the panel meeting. Those items on which the panel did reach consensus can be summarised as “keep it simple”. As with other quantitative imaging techniques, no consensus was reached with regard to the methodology for delineation of disease in WB-DWI, whether manually or by some automated/semi-automated segmentation process. Nor was there consensus on whether whole-body disease burden was preferred over a RECIST inspired approach of five target lesions as suggested by Perez-Lopez et al.^22^ By extension, there was lack of consensus on which, if any, ADC statistics should be obtained from within the delineated disease (*e.g.* standard deviation, kurtosis or skewness of ADC values within the tumour volume) or the actual tumour volume itself. The panel felt that whilst some published literature has demonstrated preliminary evidence that such statistics offer quantitative approaches for assessment of patient prognosis^24^ and heterogeneous treatment response,^25,26^ these methods are still in their infancy and required further validation before consensus could be achieved. All major manufactures provide workstation applications for *post hoc* analysis Philips Healthcare (Vistar), Siemens Healthineers (Syngo.Via), GE Healthcare (AW) and there are several third party applications OSIRIX (pixmeo, Geneva, Switzerland), Mirada Medical (Oxford, England), as well as open-source solutions such as ImageJ (National Institue for Health, USA). All provide ADC map calculations as well as ROI/VOI toolboxes and image intensity thresholding applications, as well summary statistic extraction tools. While nuclear medicine applications have been used for decades to assess quantitative 3D sectional imaging, main radiology applications such as X-ray CT and MRI have traditionally been reviewed on picture archiving and communication system (PACS) viewing stations with little or no added functionality. In truth, until this functionality has been added to PACS there will be a slow uptake in busy clinical National Health Service (NHS) imaging departments of specialty reporting tools that require additional workstations.

#### Visualisation and reporting: 

**Figure 2.**
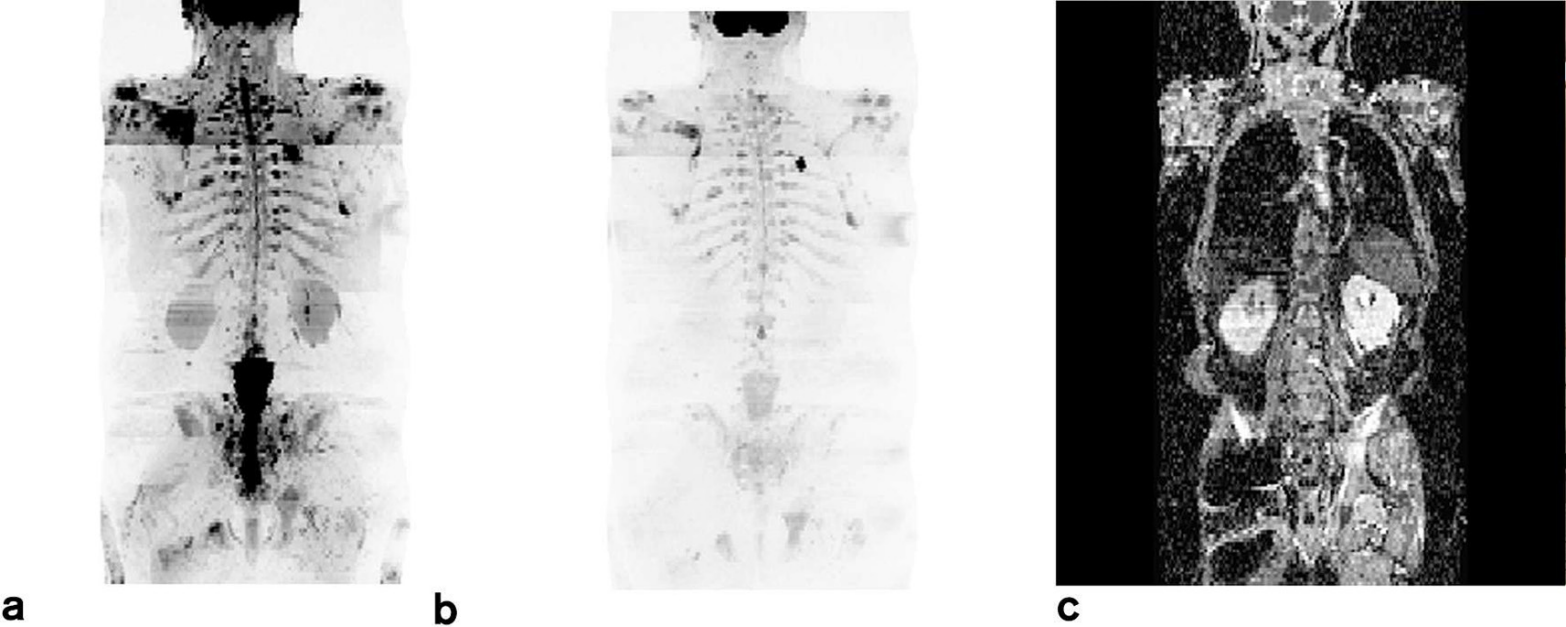
Example of a qWB-DWI data set from a patient with advanced prostate cancer (a) b = 50 s mm^–^^2^, (b) b = 900 s mm^–^^2^ and (c) corresponding ADC map calculated using a mono-exponential model. ADC, apparent diffusion coefficient; WB-DWI, whole body diffusion-weighted MRI.

The paper by Padhani et al^11^ “METastasis Reporting and Data System for Prostate Cancer: Practical Guidelines for Acquisition, Interpretation, and Reporting of Whole-body Magnetic Resonance Imaging-based Evaluations of Multiorgan Involvement in Advanced Prostate Cancer (APC)”^27^ set-out to establish minimum acceptable technical parameters for WB-MRI in APC: machine set-up, sequence specifications, routine QA/QC and radiographic aspects. Recommendations included; skull base to midthigh coverage, STIR fat suppression, 5–7 mm slice thickness, two *b*-values 50–200 and 800–1000 s mm^–^^2^, mono-exponential ADC calculation, MIP and coronal display of high *b*-value image. Although there was agreement with this guidance on WB-DWI visualisation and reporting^27–29^ on most items that overlapped; the panel did not reach consensus on the more advanced specifics of: fusion of multiparametric data sets and co-registration software functionality, and use of the inverted grey scale maximum intensity projections of the high *b*-value, etc. The panel acknowledged the recommendations for the visualisation and reporting of WB-DWI data as included in the paper by Padhani et al.^27 ^See Figure 2 for an example of a typical data set acquired on a 1.5T Siemens Magnetom scanner.

## DISCUSSION

Quantitative WB-DWI is still only available clinically in a few centres for the investigation of disseminated cancer, although numbers and publications are increasing it has yet to achieve widespread adoption. To address the challenges faced in more generalised dissemination of WB-MRI across sites, a panel of 23 experts met to provide a set of recommendations based on the consensus RAND/UCLA method on the use and acquisition of quantitative WB-DWI.

Given the increasing number of sites with both a research and clinical interest in WB-MRI within the UK; this consensus was aimed specifically at deriving a UK-specific strategy on harmonising quantitative WB-DWI. However, many of the panel members have previously taken part in international consensus panels and have an international reputation providing a non-UK centric perspective on the subject and the outcomes of a UK approach to harmonisation will also have relevance to other country/international organisations developing such guidelines.

Broadly, areas of consensus were: the need for specialist training, standardised guidelines for initial optimisation of sequence parameters, standardised routine QC tests and test objects, standardised acquisition parameters to ensure best SNR and patient comfort; the value of widespread use of quantitative WB-DWI for follow-up imaging in disseminated malignant disease; the use of the mono-exponential model for calculation of ADC; the use of summary statistics of ADC values for reporting, although ADC thresholds for “treatment response” and “disease progression” still need to be established. Consensus recommendations based on these areas are listed in Tables 1–3.

Overall, a good level of agreement was achieved for quantitative WB-DWI at 1.5T, but it was acknowledged that there are challenges of translating these protocols to 3T platforms and that more evidence from experienced users was needed. In particular, the need to understand the role of the latest technologies in optimising the DWI signal: parallel imaging, fat suppression techniques, eddy current distortion corrections and static/dynamic field inhomogeneities. Areas for which consensus were not reached (Table 5) included implementation of the latest technology and novel analysis methods associated, the exact nature of the routine QC tests for routine clinical practice and how they should be performed and how often; and the metrics used to measure, characterise (histogram and associated statistics) and visualise ADC maps.

There exists the criticism that “many imaging biomarkers, remain confined to the academic literature without real application owing to a lack of efficient and effective strategies for biomarker translation”.^12^ The European Organisation for Research and Treatment of Cancer (EORTC) position paper concluded that MRI offers a good ‘‘one size fits all’’ solution for patients who do not have substantial non-bone disease to assess therapy effectiveness^30^ and the recent interest in using MRI for radiotherapy treatment planning^31^ and the advent of MRI Linacs^32^ only highlights the need for establishing the robustness of this technique. This document has been prepared in answer to papers citing the need for comparative multimodal studies, to provide prospective quantitative data from treatment–response assessment settings.^27^ This paper describes the use of the RAND style method^17^ to achieve consensus on a range of aspects of a clinical procedure, quantitative WB-DWI, in order to obtain best practice that can be shared amongst the diagnostic radiology community in the UK. This publication should be received as a starting point for sites developing quantitative WB-DWI protocols that can then contribute to multicentre studies and enable clinical studies for specific emerging indications (*e.g.* multiple myeloma).

### Future work

The panel recognized the need to develop and promote training opportunities for radiologists, MR physicists and radiographers specifically for the implementation, QA, reporting and analysis of whole body DWI for quantitative measures.

The group expects to produce a library of useful QA procedures and tests and establish tolerances for each of these tests across scanner platforms, such that the user of a specific platform (a) can determine whether their platform is performing within acceptable tolerances in order to be able to apply quantitative whole body DWI for clinical decision-making and (b) can take remedial measures, where possible, to correct any out of tolerance results. It is the intention of the first author (supported by an NIHR fellowship) to co-ordinate and gather further data from already participating clinical trial centres^33^ to establish these tolerances.

There is also much needed, ongoing, NIHR-funded work developing post-processing techniques (*e.g.* those originally devised on *T*_1_ and *T*_2_ weighted data of the brain^34,35^ and MR manufacturer independent analysis tools to visualize ADC maps),^36^ the output of which is expected to standardize methods and help delivery of the QC tests recommended in Table 2. 

In summary, using the RAND/UCLA consensus method, a UK-based panel was able to make recommendations to provide a robust and reproducible quantitative WB-DWI protocol suitable for 1.5T to be used routinely to evaluate conditions of disseminated cancer before and after treatment. It is the panel’s intention to meet again in 3 years time to update the document.

## FUNDING

IPEM for funding travel and accommodation costs, BIR for provision of the venue. AB receives funding through HEE/NIHR Senior Lecturer fellowship (HCS SCL-2014-05-002) and is supported by the NIHR UCLH Biomedical Research Centre. SP receives funding support from the NIHR UCLH Biomedical Research Centre, KCL/UCL Comprehensive Cancer imaging Centre funded by CRUK and EPSRC and London Cancer. ST receives funding as a NIHR senior investigator and funding support from the NIHR UCLH Biomedical Research Centre, KCL/UCL Comprehensive Cancer Imaging Centre funded by CRUK and EPSRC and London Cancer. JvdM partly supported by the NIHR-CLAHRC North Thames at Bart’s Health NHS Trust. VG, GC, GCE receive financial support from the KCL/UCL Comprehensive Cancer Imaging Centres funded by CRUK and EPSRC in association with the MRC and the DoH (C1519/A16463) and from the Wellcome/EPSRC Centre for Medical Engineering at KCL (WT 203148/Z/16/Z); the DoH via the NIHR GSTT Biomedical Research Centre. AP, MG receive funding from Addenbrooke’s Charitable Trust and the NIHR comprehensive Biomedical Research Centre award to Cambridge University Hospitals NHS Foundation Trust in partnership with the University of Cambridge. DC, JW, MB, MK receive funding from CRUK and EPSRC at the Cancer Imaging Centre at ICR and RMH in association with MRC and DoH C1060/A10334, C1060/A16464 and NHS funding to the NIHR Biomedical Research Centre and the Clinical Research Facility in Imaging. AR receives funding from CRUK, NIHR (EME and HTA), NHS funding to the NIHR Biomedical Research Centre RMH and Imperial, Pelican Foundation, Imperial CRUK Centre. RP clinical fellow funded by CRUK and ECMC This report is independent research funded by the National Institute for Health Research. The views expressed in this publication are those of the author(s) and not necessarily those of the NHS, the National Institute for Health Research or the Department of Health.
